# Visualizing Global Properties of Large Complex Networks

**DOI:** 10.1371/journal.pone.0002541

**Published:** 2008-07-02

**Authors:** Weijiang Li, Hiroyuki Kurata

**Affiliations:** 1 School of Biotechnology, Southern Yangtze University, Wuxi, China; 2 The Key Laboratory of Industrial Biotechnology, Ministry of Education, Southern Yangtze University, Wuxi, China; 3 Department of Bioscience and Bioinformatics, Kyushu Institute of Technology, Kawazu, Iizuka, Fukuoka, Japan; Tel Aviv University, Israel

## Abstract

For complex biological networks, graphical representations are highly desired for understanding some design principles, but few drawing methods are available that capture topological features of a large and highly heterogeneous network, such as a protein interaction network. Here we propose the circular perspective drawing (CPD) method to visualize global structures of large complex networks. The presented CPD combines the quasi-continuous search (QCS) analogous to the steepest descent method with a random node swapping strategy for an enhanced calculation speed. The CPD depicts a network in an aesthetic manner by showing connection patterns between different parts of the network instead of detailed links between nodes. Global structural features of networks exhibited by CPD provide clues toward a comprehensive understanding of the network organizations. Availability: Software is freely available at http://www.cadlive.jp

## Introduction

Biological networks are usually large and highly heterogeneous. Intuitive representations are helpful to understand the structures of such complex and abstract objects [1]. General graph drawing methods, including those developed specifically for biological networks, are intended for explorative relational maps with all individual links clearly presented [2]–[5]. Such microscopy-oriented drawings are clearly not suitable for large complex networks, since the global features can hardly be grasped by inspecting the complex details concerning thousands of nodes connected irregularly by numerous edges.

To get macroscopic views of a complex network, a possible way is to divide the original network into smaller parts and then display the relationships between these parts. However, this is usually not feasible for biological networks such as protein interaction networks (PINs) because they do not contain naturally separated parts and how to divide them remains to be solved.

A circular perspective drawing (CPD) method often illustrates plain architectures of a graph network, such as regular, random, and small-world networks, while CPD is not employed for explorative visualization of large complex networks because numerous tangled edges would fill the whole circle and make links undistinguishable. Consequently, its applications are limited to small networks and no effective algorithm is available for large networks. Existing techniques have high computation demands and thus unsuitable for large networks [6]. In this work, we propose a new efficient circular layout algorithm capable of computing satisfied layouts for large networks with thousands of nodes in an aesthetic drawing manner. The presented CPD algorithm combines the quasi-continuous search (QCS) analogous to the steepest descent method with a random node swapping strategy for an enhanced calculation speed. The CPD method depicts edge distribution on a disc around which the nodes sit uniformly in an optimized order that minimizes edge lengths. It greatly facilitates an intuitive or visual understanding of topological features of a large-scale complex network.

## Methods

### Circular perspective drawing method

A CPD is determined by the node placement, also known as circular layout, which can be expressed as a circular permutation **p** = (*p*_1_, *p*_2_, …, *p_n_*) of (1, 2, …, *n*), *n* = the number of nodes. The *i*th node is placed at position 
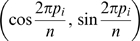
. The goal of node placement is to show topological proximity between nodes by geometric positions such that nodes of densely connected subnets tend to appear in near places. This is achieved by minimizing the objective function
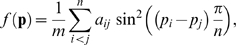
(1)where *m* is the number of edges. If there is a link connecting nodes *i* and *j*, *a_ij_* = 1; otherwise, 0. It is easy to verify that 4*f*(**p**) is equal to the mean squared edge lengths and 0≤*f*≤1.

Usually circular layout methods try to minimize the number of edge crossings. Our goal is to find favorable global configurations and detailed positions geometric neighboring nodes are of minor concern, so minimizing edge lengths is more appropriate. In addition, techniques employed for crossing reduction are based on systematic swapping of node positions [6], whose *O*(*m* ^2^) time requirement for one local minimum makes their applications to PINs impractical.

Here we propose a new method that is able to efficiently find satisfactory circular layouts for large complex networks. The main minimization algorithm of our method, called quasi-continuous search (QCS), is analogous to the steepest descent method. We first calculate the steepest descent direction of *f*(**p**),
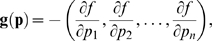
(2)where **p** is temporarily seen as a continuous vector. In continuous steepest descent methods, the search point is given by
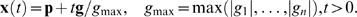
(3)For our purpose, **x**(*t*) needs to be discretized to represent a valid circular layout; otherwise all nodes will aggregate at a same position. So we set the search point **p**(*t*) = (*p*_1_(*t*),…,*p_n_*(*t*)) by discretizing **x**(*t*) = (*x*_1_(*t*),…,*x_n_*(*t*)) through letting
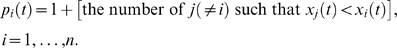
(4)In other words, *p_i_*(*t*) is the rank of *x_i_*(*t*) in the ascendingly ordered sequence of {*x*_1_(*t*),…,*x_n_*(*t*)}.

For an input layout **p**, a better layout can be found by comparing a series of candidate layouts **p**(*t*) with different *t*. At the end of QCS, the layout **p** is updated to the best candidate **p**(*t*_0_) such that *f*(**p**(*t*_0_)) is minimal. To avoid being trapped in local minima, search points are set in a large range along the steepest-descent direction with *t* = *t*_max_, *t*_max_/2, *t*_max_/4, until *t*_min_. Numerical experiments show that *t*_max_ = *n*/5 and *t*_min_ = 3 are choices suitable for all tested cases.

After a QCS, most nodes are placed near their correct positions but there is still space to further optimize the configuration through swapping node positions, because node swapping is generally hard to be realized by QCS. We use a random swapping strategy to avoid the inefficiency of systematic swapping: In each trial, a pair of nodes are randomly chosen and exchange their positions if the trial hits, i.e., the exchange lowers down the *f* value. As the optimization proceeds, a hit needs more trials and the procedure becomes less efficient. Therefore the random swapping will be stopped when either a certain number of hits (say, *R* = 50) are found or a large number of trials (say, *S* = 100*n*) are tested. The optimization is then switched to QCS again. The whole process is sufficiently repeated until no hit is found in a random swapping phase. For clarity, the algorithm is outlined in Table 1. The application program is written in Matlab (The Mathworks, Inc.) (Program S1).

**Table 1:** Circular layout algorithm.

1	Form a random layout
2	Repeat at most *R*_max_ times
3	Optimize the current layout using QCS
4	Repeat at most *S*_max_ times
5	Randomly select a pair of nodes, swap their positions if the *f* value is decreased;
6	If *S*_min_ swappings have been found in this cycle then exit this repeat cycle
7	End repeat
8	If no hit is found in above cycle then terminate
9	End repeat

Extensively numerical experiments show that the control parameters *R*_max_ = 500, *S*_max_ = 100*n*, and *S*_min_ = 50 are suitable for tested networks with up to 10000 nodes. To achieve further high quality, we used a simple best-of-five strategy: calculating 5 layouts and picking the best one (with the lowest *f* value) as the output.

### Drawing method

For a large complex network, an effective drawing method is necessary to make the circular layout visually meaningful. Instead of directly plotting the edges, CPD shows an image that displays how the edges distribute on the disc enveloped by the layout circle. A pixel of the CPD image represents to a small grid square on the disc, whose color is determined by calculating the edge count, i.e. the number of edges that pass through the corresponding grid square. Edge distributions can be shown by properly setting the functional relation between the color and the edge count. Of course, there are many suitable color mappings. We find that satisfactory visual effects can usually be obtained through color maps with brightness scaled to logarithm of the edge count. Here a CPD is produced by displaying the matrix log(1+*c*) as an image using the MATLAB “bone” color map, where *c* is the matrix whose elements are edge counts of the corresponding pixels; the log function operates element-wise on 1+*c*.

### Assessment of the drawing algorithm

Quality of a CPD is determined by the node placement. Each run of the algorithm yields a different CPD due to the initial random layouts and the random node swapping steps. Are suboptimal solutions found by the algorithm close enough to the absolute optimum? The *f* value itself is not adequate to answer this question because the absolute minimum is unknown. So we define similarity score between two circular layouts **p** and **p**′
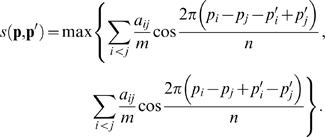
(5)Obviously, −1≤*s*≤1 and for two equivalent or mirror-equivalent layouts, *s* = 1. The score describes similarity between detailed structures of two layouts in terms of relative configurations between nodes and their partners. Higher scores imply more similar detailed relative configurations. Conversely, uncorrelated layouts have similarity scores around 0.

### The yeast PIN

**Figure 1:**
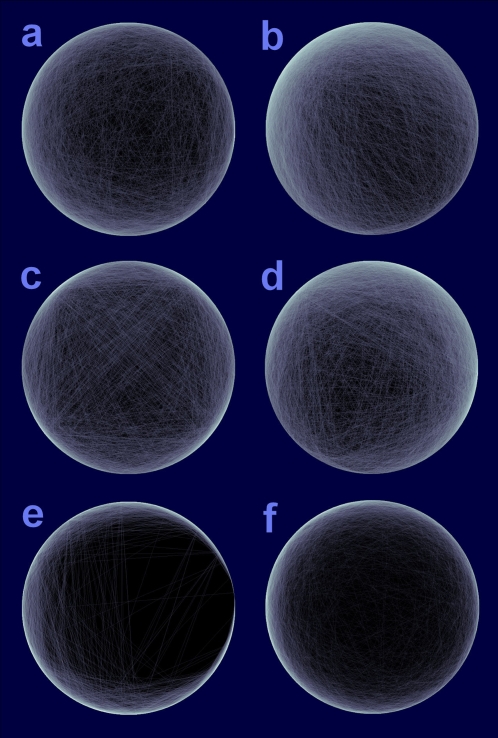
Global views of protein interaction networks by circular perspective drawings. Each disc image represents a network whose nodes sit uniformly along the perimeter with minimal edge lengths. Pixels of the images are rendered colors with brightness proportional to logarithm of the number of passing-through edges. Nodes with only one link or disconnected to giant components were ignored in producing the drawings. See Materials and Methods for details of the networks. a, The yeast PIN. b, A randomized yeast PIN. c, A fully clusterized yeast PIN. The 4 clusters are clearly shown by the bright regions. d, A partially clusterized yeast PIN with a neutral group. e, A DD model of the yeast PIN. f, A DDR model of the yeast PIN.

The yeast PIN data used in this research was taken from DIP by removing multiple links and self-loops [7]. The original yeast PIN consists of 4625 nodes belonging to 50 disconnected components. The giant component contains 4524 nodes, 1177 of them are leaf nodes having only single links. The leaf nodes were excluded because they have little influence on global connection patterns but they interfere with the whole views. The resulting network (shown in Fig. 1a) contains 3347 nodes and 12672 edges (7.6 links per node on average).

### Network randomization

A network is randomized by sufficiently rewiring the edges. In each trial of rewiring, a pair edges are randomly chosen. The edges exchange one of their incident nodes unless the exchange creates an edge already present in the network or a self-loop. The trial is repeated 500*m* (*m* = number of edges) times. On completion, each edge is rewired about 1000 times on average. (Note that two edges are rewired in each successful trial.) Figure 1b shows the giant component (excluding leaf nodes) of a randomized yeast PIN.

### Network clusterization

A network is clusterized by biased edge-rewiring similar to the randomization procedure but some rewirings may be suppressed if they result in unfavorable edges which connect nodes from different cohesive groups. Specifically, if a valid rewiring increases the number of unfavorable edges by *k*, the rewiring is realized with probability *w*(*k*),
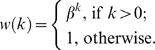
We use *β* = 0.1 and perform 10*m* rewiring trials to generate fully or partially clusterized networks.

A fully clusterized yeast PIN is generated by a biased edge-rewiring with the nodes randomly classified into 4 cohesive groups. After the clusterization, most edges connect nodes within the same groups and thus the resulting network has 4 clusters, whose giant component excluding leaf nodes is shown in Fig. 1c.

A partially clusterized yeast PIN is generated with the nodes randomly classified into 4 groups, three cohesive and one neutral. In the process of rewiring, only edges between different cohesive groups are unfavorable; edges starting from the neutral group have no preferential targets. So the resulting network has partial clusters. The giant component excluding leaf nodes is shown in Fig. 1d.

### The duplication-divergence (DD) model of yeast PIN

A DD model network is generated by mimicking an evolution process with gene duplication and function divergence [8], [9]. Starting from three connected nodes, the network grows through node cloning: At each time step, a randomly chosen node is duplicated to produce a new node. Each link of the original node is copied with a probability *r* to the cloned node. If the cloned node has no link, it is removed from the network. We use *r* = 0.45 and choose a generated network that has 4625 nodes and best fits the degree sequences of the original yeast PIN (with 4625 nodes). The degree distribution of the model network is very similar to that of the yeast PIN. The resulting giant component excluding leaf nodes is shown in Fig. 1e.

### The duplication-divergence-with-random-mutation (DDR) model of yeast PIN

The DDR model is similar to the DD model except a mechanism to produce new function: In addition to inheriting (with probability *r*) links from the original node, the cloned node has a probability *q* in each time step to develop a new link with an existing node. We use *r* = 0.38, *q* = 0.56 and generate a network with 4625 nodes and almost the same number of connections as the original yeast PIN (with 4625 nodes), although the degree distribution of the generated network is significantly different from that of the yeast PIN's. The giant component excluding leaf nodes is shown in Fig. 1f.

## Results and Discussion

Protein interaction networks (PINs) are a typical kind of complex biological networks. Extensive studies discovered various structural properties with important biological implications[10]–[13], whereas few methods can intuitively show how the proteins organizes into such intricate structures. Here we used the yeast PIN and its variants to demonstrate what global properties can be visualized through CPD as shown in Fig. 1.

A bright narrowband around the circle is an outstanding feature displayed by CPD of the yeast PIN (Fig. 1a): Most links are short and connect closely sitting nodes, which means that most nodes have a narrow spectrum of partners. Such locality does not divide the network into obviously separated clusters, forming a giant component, whereas all nodes are well interconnected through local links. In CPD, edge distribution is represented by colors: A brighter region means there are more edges passing through it. Another important property revealed by the CPD is disassortative mixing, i.e., hubs (highly connected nodes) tend to connect low degree partners [12], [13], which results in the continuous bright ring distributed evenly around the whole circumference. Otherwise hubs will aggregate together and show concentrated brightest regions as shown in Figs.1b–d, which shows the differences between the yeast PIN and its biased-randomized variants (See Methods). In Fig. 1b the concentrated brightest region is identified on the top left, where some hubs are aggregated. In Fig. 1c four brightest regions are seen clearly, while partial clusters (Fig. 1d) are harder to detect than the four clusters (Fig 1c) because of the overlapping caused by the neutral group [14]. Noting that the background networks of Figs. 1a–d have exactly the same degree distribution, we see the large freedom for network structure to vary under the constraint of degree distribution.

Evolution-inspired models shed lights on inferring mechanisms from experimental PIN data. They were basically tested by the ability to reproduce the degree distribution of target PINs. With CPD, we can check the models comprehensively and intuitively. Figure 1e is a network generated by the duplication-divergence (DD) model, which is the simplest model that has only one control parameter. A bright narrowband is seen around the circle, although it becomes very thin in the right. This indicates a similar global connection pattern between the yeast PIN (Fig. 1a) and the DD model (Fig. 1e), where a giant component exists. The DDR model, a modified DD model that adds a mechanism for nodes to develop new random connections, has a more similar look to the yeast PIN (Fig. 1f) [8], [9]. A continuous narrow ring distributed evenly appears around the whole circumference, where a giant component exists while hubs are not aggregated so much (disassortative mixing). This suggests that new interactions produced by random mutations contribute significantly to the whole PIN topology. In comparison with the topology of several typical networks, special characteristics of the yeast PIN can be captured by using CPD.

We characterized the CPD algorithm, where 100 circular layouts were calculated for the yeast PIN with 3347 nodes and 12672 edges (a giant component excluding leaf nodes). The average time for one layout was 53s on a Pentium IV 2.8 GHz desktop computer, showing the very fast calculation speed. All the layouts had approximately equaled *f* values: *f* = 0.248±0.002 (standard deviation). The similarity score between any two layouts was *s* = 0.77±0.06. This shows that the proposed CPD method finds near optimal layouts with very similar global configurations and therefore produces consistent drawings for same networks.

So far several circular layout algorithms have been developed to distribute network nodes on a ring based on various measures such as edge crossings, compactness and deviation angles, while they have not intensively pursued the application to a large-scale network with thousands of nodes, because the circular ring is too narrow to accommodate a huge number of nodes[6], [15], [16]. Compared with such existing methods, the outstanding features of the presented CPD are that it draws a large-scale network with thousand of nodes by employing a fast QCS algorithm that efficiently minimizes the edge lengths and that it provides an intuitive or global understanding for intricate structures of large-scale networks, such as modular, DD and DDR structures. These features of CPD are due to the fact that the overall distribution of the edges is a major factor to layout the network instead of the node location.

## Supporting Information

Program S1 The CPD program in written in Matlab (ZIP file).
(0.07 MB ZIP)
